# Altered Hippocampal Activation in Seizure-Prone *CACNA2D2* Knock-out Mice

**DOI:** 10.1523/ENEURO.0486-23.2024

**Published:** 2024-05-03

**Authors:** Alyssa B. Danis, Ashlynn A. Gallagher, Ashley N. Anderson, Arielle Isakharov, Kathleen A. Beeson, Eric Schnell

**Affiliations:** ^1^Department of Anesthesiology and Perioperative Medicine, Oregon Health & Science University, Portland, Oregon 97239; ^2^Research and Development Service, Portland VA Health Care System, Portland, Oregon 97239; ^3^Neuroscience Graduate Program, Oregon Health & Science University, Portland, Oregon 97239

**Keywords:** alpha-2-delta, calcium channels, dentate gyrus, epilepsy, hippocampus

## Abstract

The voltage-gated calcium channel subunit α2δ-2 controls calcium-dependent signaling in neurons, and loss of this subunit causes epilepsy in both mice and humans. To determine whether mice without α2δ-2 demonstrate hippocampal activation or histopathological changes associated with seizure activity, we measured expression of the activity-dependent gene c*-fos* and various histopathological correlates of temporal lobe epilepsy (TLE) in hippocampal tissue from wild-type (WT) and α2δ-2 knock-out (*CACNA2D2* KO) mice using immunohistochemical staining and confocal microscopy. Both genotypes demonstrated similarly sparse c-*fos* and *ΔFosB* expressions within the hippocampal dentate granule cell layer (GCL) at baseline, consistent with no difference in basal activity of granule cells between genotypes. Surprisingly, when mice were assayed 1 h after handling-associated convulsions, KO mice had fewer c-*fos*-positive cells but dramatically increased *ΔFosB* expression in the dentate gyrus compared with WT mice. After administration of a subthreshold pentylenetetrazol dose, however, KO mice dentate had significantly more c-*fos* expression compared with WT mice. Other histopathological markers of TLE in these mice, including markers of neurogenesis, glial activation, and mossy fiber sprouting, were similar between WT and KO mice, apart from a small but statistically significant increase in hilar mossy cell density, opposite to what is typically found in mice with TLE. This suggests that the differences in seizure-associated dentate gyrus function in the absence of α2δ-2 protein are likely due to altered functional properties of the network without associated structural changes in the hippocampus at the typical age of seizure onset.

## Significance Statement

Calcium channel α2δ subunits play important roles in controlling neuronal circuit structure and function, and mutation of the α2δ-2 isoform of this protein is associated with spontaneous seizures. In this study, we found that seizures in α2δ-2 mutant mice involve altered activation of the hippocampal dentate gyrus without substantial histopathological changes in the hippocampal structure. This suggests that the differences in seizure-associated dentate function are likely due to altered functional properties of the network rather than gross changes in anatomical circuit organization.

## Introduction

Mutations in ion channel genes can dramatically alter neuronal function and in severe cases cause genetic forms of epilepsy known as channelopathies (Davies and Hanna, 1999). In particular, voltage-gated calcium channels (VGCCs) play critical roles in controlling neuronal excitability, synaptic transmission, and synaptic plasticity, and mutations in many VGCC genes cause treatment-resistant forms of epilepsy that can begin neonatally (Catterall, 2011). These syndromes are sometimes associated with structural brain defects, which could alter neuronal circuit connectivity and thus secondarily contribute to circuit hyperexcitability (Rajakulendran and Hanna, 2016).

Although channelopathies often result from mutations in pore-forming subunits that critically alter channel properties, mutations in non-pore-forming auxiliary VGCC subunits also cause genetic forms of epilepsy. One such subunit is the α2δ-2 protein, encoded by the *CACNA2D2* gene, originally associated with a heritable form of epilepsy in the spontaneous α2δ-2 mutant *ducky* mouse (Snell, 1955; Barclay et al., 2001). Subsequent work identified additional spontaneous mutant *CACNA2D2* alleles in mice (Brill et al., 2004), and epileptic phenotypes have been associated with both targeted mutations of *CACNA2D2* and spontaneous *CACNA2D2* mutations in humans (Ivanov et al., 2004; Edvardson et al., 2013; Pippucci et al., 2013).

Although the α2δ-2 subunit does not notably alter VGCC voltage dependence or conductance, it plays a significant role in the surface and subcellular trafficking of VGCCs, which could dramatically alter neuronal circuit structure and function (Barclay et al., 2001). Additionally, a separate member of the α2δ family of proteins, the α2δ-1 isoform, is a neuronal thrombospondin receptor with synaptogenic functions, demonstrating that α2δ subunits can have signaling roles independent of VGCCs that could alter neuronal circuits (Eroglu et al., 2009). However, despite the known and hypothesized cellular roles of α2δ-2, the specific circuit alterations in *CACNA2D2* mutant mice that contribute to seizure initiation and propagation are unknown.

*CACNA2D2* mutant mice exhibit handling-associated convulsions beginning at an early age, and electroencephalographic spike-wave discharge (SWD) activity and absence-type seizures in young adult mice, which can progress to generalized seizures strongly associated with high juvenile mortality (Snell, 1955; Meier, 1968; Barclay et al., 2001). SWDs are typically associated with aberrant thalamocortical activation (Blumenfeld, 2005). However, as α2δ-2 protein is expressed in the hippocampus (Cole et al., 2005; Lein et al., 2007) and SWDs typically are associated with behavioral arrest rather than convulsions (Barclay et al., 2001), we wondered whether structural or functional alterations in the hippocampus might be associated with, and potentially contribute to, the progression to generalized seizure activity in *CACNA2D2* mutant mice. We focused primarily on the dentate gyrus and CA3 hippocampal subfields, as these regions have numerous well-characterized histopathological abnormalities in epilepsy and can serve to gate seizure activity (Krook-Magnuson et al., 2015; Scharfman, 2019). To investigate a potential relationship between *CACNA2D2*-associated epilepsy and structural brain changes associated with temporal lobe epilepsy (TLE), we examined hippocampal sections from WT and *CACNA2D2* KO mice for patterns of granule cell activity based on the expression of the activity-dependent immediate early genes c-*fos* and *ΔFosB*, as well as for a range of other histopathological markers typically associated with TLE.

## Materials and Methods

### Mice

*CACNA2D2* KO mice (*CACNA2D2^tm1Svi^*, MGI:3055290, RRID:MGI:3055322) were created by homologous recombination and maintained on a C57BL/6 background (Ivanov et al., 2004). Mice were housed in an AAALAC-certified facility, and all animal procedures were performed in accordance with the Portland VA Health Care System's Institutional Animal Care and Use Committee's regulations. Mice were placed on a 12 h light/dark cycle with continuous access to food/water *ad libitum*, and experiments/harvests were performed at Zeitgeber time ZT4–6 (4–6 h after lights on). This strain was maintained by breeding heterozygotic mice, which produced WT and KO littermates for experimental analysis. All mice were genotyped by PCR as previously described (Beeson et al., 2020), and all KOs were additionally verified by morphological phenotype, which consisted of small size, hindlimb clasping, and ataxic gait. Mice were group housed with same-sex littermates of mixed genotype at weaning.

WT and KO mice of both sexes were analyzed at 4–5 weeks of age. In a majority of cases, each mouse was paired with a littermate of the converse genotype. For some litters, however, a Mendelian ratio was not achieved, so mice were paired across litters when necessary to balance genotypes. Given the high mortality of mice at weaning age (∼28 d old) with conventional husbandry (Meier, 1968), we adapted our weaning protocols to provide soft food continuously after weaning, with gentle handling, as previously shown to improve survival (Geisler et al., 2021). Although this study was not powered to detect sex differences, male and female mice did not demonstrate any sex differences in any of the activity-dependent labeling and seizure assays (see Table 1 for comparisons between sexes in each group) and were combined for all analyses between genotypes.

**Table 1.** Statistical comparison between males and females for datasets

Comparison	Data structure	Statistical test	Difference between means	Power (95% confidence intervals)
WT M vs F				
Baseline c-*fos*+ total cell density	Non-normal (*W* = 0.7405)	Mann–Whitney test	0.7857	Rank sums 24 vs 12
Baseline c-*fos*+ total cell density (including outlier)	Non-normal (*W* = 0.7405)	Mann–Whitney test	0.7857	Rank sums 24 vs 12
Baseline c-*fos*+ semi-lunar cell density	Normal (0.9474)	Unpaired *t* test	0.9296	−697.0 to 751.5
Baseline c-*fos*+ granule cell density	Non-normal (*W* + 0.7551)	Mann–Whitney test	0.3929	Rank sums 26 vs 10
Handling seizure score	*t* test invalid since all values were the same	Mann–Whitney test	>0.9999	Rank sums 5 vs 5
Handling c-*fos*+ cell density (WT vs KO)	*n* too small	Mann–Whitney test	0.3333	Rank sums 3 vs 7
PTZ seizure score	Non-normal (*W* = 0.6840)	Mann–Whitney test	0.625	Rank sums 20.50 vs 15.50
PTZ c-*fos*+ cell density	Non-normal (*W* = 0.6676)	Mann–Whitney test	>0.9999	Rank sums 22 vs 14
Baseline VENTRAL c-*fos* density	Non-normal (*W* = 0.7507)	Mann–Whitney test	>0.9999	Rank sums 10 vs 11
PTZ VENTRAL c-*fos* density	Non-normal (*W* = 0.7304)	Mann–Whitney test	0.7857	Rank sums 24 vs 12
Handling VENTRAL c-*fos* density	*n* too small	Unpaired *t* test	0.2830	−5,498 to 11,114
Baseline ΔFosB density	Normal (*W* = 0.8406)	Unpaired *t* test	0.9736	−473.5 to 486.5
PTZ ΔFosB density	Normal (*W* = 0.7964)	Unpaired *t* test	0.7861	−341.1 to 430.6
Handling ΔFosB density	*n* too small	Mann–Whitney test	>0.9999	Rank sums 5 vs 5
Calretinin+ cell density	Normal (*W* = 0.8175)	Unpaired *t* test	0.2254	−200.7 to 57.89
CA3 pyramidal cell layer thickness	Normal (*W* = 0.8107)	Unpaired *t* test	0.33	−49.22 to 114.6
CA3 pyramidal cell density	Normal (*W* = 0.8614)	Unpaired *t* test	0.5758	−193.0 to 301.3
Mossy fiber sprouting	Non-normal (*W* = 0.7500)	Mann–Whitney test	>0.9999	Rank sums 11 vs 10
Ki67+ cell density	Normal (*W* = 0.8941)	Unpaired *t* test	0.3822	−18,299 to 38,292
DCX intensity	Normal (*W* = 0.8491)	Unpaired *t* test	0.7252	−1,094 to 832.1
PV+ cell density in the GCL	Normal (*W* = 0.9792)	Unpaired *t* test	0.8369	−1,212 to 1,421
PV+ cell density in the hilus	Normal (*W* = 0.9130)	Unpaired *t* test	0.2856	−222.1 to 576.1
PV+ cell density in CA3	Non-normal (*W* = 0.7626)	Mann–Whitney test	0.7	Rank sums 12 vs 9
GFAP+ cell density	Normal (*W* = 0.9643)	Unpaired *t* test	0.4045	−6,499 to 13,058
GFAP intensity	Normal (*W* = 0.9075)	Unpaired *t* test	0.6164	−100.5 to 67.64
Iba1 cell density	Normal (*W* = 0.7789)	Unpaired *t* test	0.2115	−122.6 to 37.11
KO M vs F				
Baseline c-*fos*+ total cell density	Normal	Unpaired *t* test	0.9984	−6,321 to 6,331
Baseline c-*fos*+ total cell density (including outlier)	Non-normal (*W* = 0.6249)	Mann–Whitney test	>0.9999	Rank sums 18 vs 18
Baseline c-*fos*+ semi-lunar cell density	Normal (*W* = 0.7743)	Unpaired *t* test	0.4724	−2,277 to 1,220
Baseline c-*fos*+ granule cell density	Normal (*W* = 0.8128)	Unpaired *t* test	0.7769	−4,053 to 5,121
Handling seizure score	*n* too small	Unpaired *t* test	0.6667	−3.202 to 2.535
Handling c-*fos*+ cell density (WT vs KO)	*n* too small	Unpaired *t* test	0.7693	−8.118 to 6.944
PTZ seizure score	Non-normal (*W* = 0.6298)	Mann–Whitney test	>0.9999	Rank sums 18.50 vs 17.50
PTZ c-*fos*+ cell density	Normal (*W* = 0.7855)	Unpaired *t* test	0.5879	−128,579 to 207,079
Baseline VENTRAL c-*fos* density	Non-normal (*W* = 0.6567)	Mann–Whitney test	0.5333	Rank sums 12 vs 9
PTZ VENTRAL c-*fos* density	Non-normal (*W* = 0.7307)	Mann–Whitney test	0.1143	Rank sums 12 vs 24
Handling VENTRAL c-*fos* density	*n* too small	Unpaired *t* test	0.7993	−8,964 to 7,833
Baseline ΔFosB density	Non-normal (*W* = 0.6446)	Mann–Whitney test	>0.9999	Rank sums 16 vs 12
PTZ ΔFosB density	Non-normal (*W* = 0.6819)	Mann–Whitney test	0.6857	Rank sums 16 vs 20
Handling ΔFosB density	*n* too small	Unpaired *t* test	0.8803	−1,395 to 1,510
Calretinin+ cell density	Normal (*W* = 0.9085)	Unpaired *t* test	0.028	22.99 to 281.9
CA3 pyramidal cell layer thickness	*n* too small	Unpaired *t* test	0.9133	−68.89 to 74.90
CA3 pyramidal cell density	*n* too small	Unpaired *t* test	0.4075	−176.6 to 353.1
Mossy fiber sprouting	*n* too small	Unpaired *t* test	0.3552	−0.6859 to 0.3109
Ki67+ cell density	*n* too small	Unpaired *t* test	0.4626	−45,218 to 24,765
DCX intensity	*n* too small	Unpaired *t* test	0.772	−989.4 to 790.7
PV+ cell density in the GCL	*n* too small	Unpaired *t* test	0.0969	−1,858 to 232.3
PV+ cell density in the hilus	*n* too small	Unpaired *t* test	0.1567	−396.1 to 90.80
PV+ cell density in CA3	*n* too small	Unpaired *t* test	0.1226	−17,245 to 3,005
GFAP+ cell density	*n* too small	Unpaired *t* test	0.8673	−8,163 to 7,179
GFAP intensity	*n* too small	Unpaired *t* test	0.3264	−125.3 to 53.38
Iba1 cell density	*n* too small	Unpaired *t* test	0.8081	−40.60 to 48.97

### Acute seizure modeling

Pentylenetetrazol (PTZ; Sigma) was diluted in sterile water at 3 mg/ml and administered to a subset of mice by intraperitoneal injection at a dosage of 30 mg/kg. Mice were continually observed for seizures for 1 h after administration, which were scored on a modified Racine scale for mice (Shibley and Smith, 2002), with a zero score given for normal behavior without any tremor or freezing and a six for high-amplitude jumping/popcorn behavior. PTZ injection in WT mice (at higher doses) typically causes a short-latency (5–10 min) self-limited seizure episode (1–5 min) with no status epilepticus or recurrent events. After 1 h, mice were humanely killed by anesthetic overdose for brain harvest.

To assess the hippocampal involvement in handling-induced seizure activity (Snell, 1955; Meier, 1968), we manually removed a separate cohort of WT and KO mice from their cages by a gloved investigator by the base of the tail, rotated at 180°, and placed on a wire cage top. They were then picked up using a gentle scruffing technique and held in the supine (belly up) position for 5 s. After this maneuver, mice were returned to their home cages for 1 h of observation and seizure scoring prior to killing for histologic analysis.

### Histology

Prior to brain harvest, mice were killed under deep isoflurane/avertin anesthesia by transcardiac perfusion and decapitation in accordance with IACUC-approved protocols. Transcardiac perfusion consisted of 5 ml of phosphate-buffered saline (PBS), pH 7.4, followed by 10 ml of 4% paraformaldehyde in PBS (PFA). After dissection, brains were postfixed overnight in PFA, rinsed thrice in PBS, and cut into 100-µm-thick coronal sections on a Leica VT1000 Vibratome. Dorsal brain sections were selected for staining beginning three sections posterior to the appearance of the hippocampus bilaterally in each section, corresponding to approximately −1.6 mm posterior to bregma in the adult atlas, and extending to −2.0 mm posterior to bregma for dorsal c-*fos* staining and kept consistent between mice.

Free-floating brain sections were permeabilized and blocked in 0.4% Triton X-100 in PBS (PBST) containing 10% goat serum for 1 h and stained overnight at 4°C with the following antibodies diluted in PBST + 1.5% goat serum: rabbit anti-c-fos, Cell Signaling 2250, RRID:AB_2247211, 1:300; rabbit anti-ΔFosB, Cell Signaling 14695, RRID:AB_2798577, 1:400; guinea pig anti-ZnT3, Synaptic Systems 197004, RRID:AB_2189667, 1:200; mouse anti-PV, NeuroMab 75-455, RRID:AB_2629420, 1:20; goat anti-calretinin, Swant CG1, RRID:AB_10000342, 1:500; rabbit anti-Ki67, Millipore AB9260, RRID:AB_2142366, 1:500; guinea pig anti-doublecortin (Dcx), Millipore AB2253, RRID:AB_1586992, 1:500; mouse anti-glial fibrillary acidic protein (GFAP), Sigma G3893, RRID:AB_477010, 1:500; rabbit anti-Iba1, Wako 019-19741, RRID:AB_839504, 1:1000; rat anti-Mac-2, Cedarlane CL8942AP, RRID:AB_10060357, 1:1000. The following morning, the slices were rinsed in PBST thrice for 5 min each and incubated in Alexa Fluor-conjugated secondary antibodies (goat or donkey, Alexa 488/568/647, 1:400 each, Invitrogen) in PBST + 1.5% goat serum at room temperature for 4–6 h. Sections were subsequently rinsed, counterstained with DAPI 1:20,000 for 10 min and mounted on glass slides with Fluoromount-G. For anti-*c-fos*, anti-ΔFosB, anti-Ki67, anti-Dcx, and anti-GFAP stains, the slices underwent an antigen retrieval step at 95°C (for 15 min, followed by gradual cooling per the manufacturer's instructions; DAKO) prior to permeabilization and blocking to improve antigenicity.

### Imaging and analysis

Slides were imaged on an upright Scientifica SliceScope with Olympus optics, using 10× 0.4NA, 20× 0.8NA, and 40× 1.4NA objectives, and acquired with a Photometrics 95 camera through a Yokogawa CSU-W1 spinning disk confocal system running SlideBook software (Intelligent Imaging). After acquisition, images were transferred to a separate computer for manual counting/analysis using FIJI/ImageJ software. WT and KO sections were stained side-by-side with identical solutions for each assay. At mounting, all slides were coded to deidentify animal ID/genotype prior to imaging, and all imaging, image analysis, and quantification were performed by an investigator blinded to animal/slide identity/genotype. The results were unblinded and combined by a third investigator only after analysis and quantification was complete. Cell densities were quantified using either single confocal sections or confocal image *Z*-stacks, as detailed for each specific experiment below. Images were acquired from up to four hippocampal sections per animal for each experiment; sections were excluded (prior to unblinding) if the section was damaged during mounting or an air bubble obscured tissue visualization.

*c-fos*+ cell densities in the dorsal dentate gyrus were calculated from confocal image stacks acquired with a 10× objective from four sections per mouse in most cases, although a small number of individual images were discarded prior to quantification/unblinding if technical issues (air bubbles, tissue folding during mounting, missing/damaged slices) precluded the quantification from any individual image. For each mouse, *c-fos*+ cell densities from each image were averaged to obtain a single value per animal. All *c-fos*+ cells in the granule cell layer (GCL) or at the GCL/inner molecular layer (IML) border were counted and normalized to the GCL volume in the image stack. GCL volume (in mm^3^) was calculated as the GCL area determined by tracing DAPI-stained granule cell nuclei × depth of stack. To subcategorize putative semilunar granule cells, cells that were outside of the GCL but within 10 µm (approximately one granule cell soma diameter) of the GCL–IML border were counted separately and normalized to the same GCL volume. Total *c-fos*+ cell counts represent the combined cell counts of putative granule cells and semilunar cells in each group; subcategorized data can be found in the text. *ΔFosB* staining fluorescence intensity was graded between granule cells (much less binary than c-fos) and thus quantified by measuring the average fluorescence intensity of *ΔFosB*-stained tissue within the GCL, minus the level of background staining, from a 10× confocal section imaged near the surface of the slice, using identical side-by-side staining and imaging protocols. One pair of samples was lost between the c-*fos* and *ΔFosB* staining, resulting in *n* = 7 rather than *n* = 8 per baseline group for this analysis.

**Figure 1.**
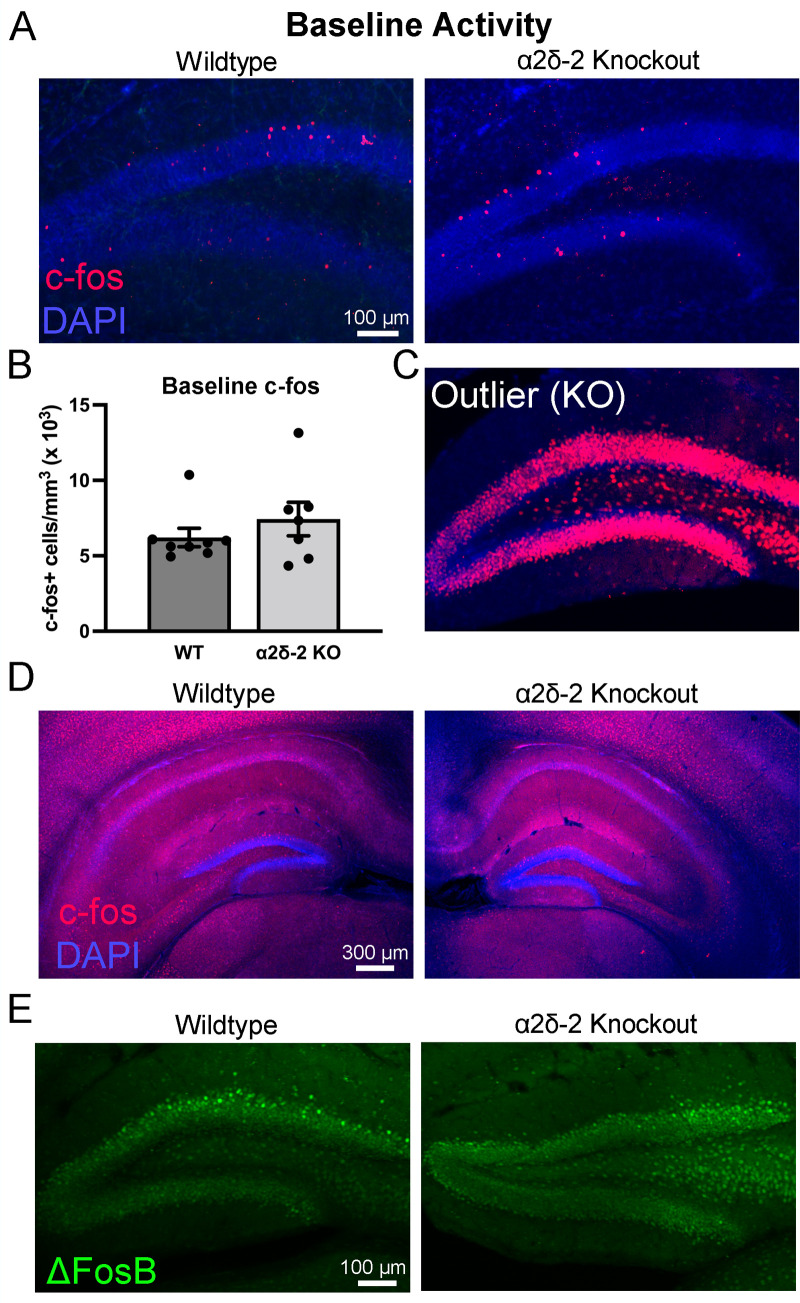
Granule cell expression of the activity-dependent genes c-*fos* and Δ*FosB*. ***A***, Example images from 4-week-old WT and *CACNA2D2* KO mouse hippocampal sections, demonstrating c-*fos*+ granule cells (red) sparsely located throughout the GCL and near the granule cell–molecular layer border. Cell nuclei were visualized using DAPI stain (blue). ***B***, Quantification of c-*fos*+ cell density (*n* = 8, 7; *p* = 0.40). ***C***, One KO mouse excluded from baseline analysis, due to global dentate c-*fos* expression, suggestive of recent seizure. ***D***, Lower power images of c-*fos* staining for WT and KO mice. ***E***, Representative Δ*FosB* expression (green) in WT and *CACNA2D2* KO dentate gyrus in mice at baseline.

Hilar mossy cell density was quantified as the number of calretinin-positive cells in the hilar area [bounded by the subgranular zone (SGZ) and edges of the image] divided by the hilar area imaged in two dimensions, from a single confocal section acquired at 20×. Small (<10 µm diameter) calretinin-positive cells located within the GCL or within 10 µm of the inner edge of that layer (SGZ) were considered to be immature granule cells (Brandt et al., 2003) and not quantified. Parvalbumin-positive (PV+) cell density was quantified as the number of PV+ cells divided by the volume of the GCL or CA3 pyramidal cell layer imaged for measurements made in the dentate or CA3, respectively. CA3 pyramidal cell layer width was defined as the average transverse width of the cell layer (defined using DAPI+ nuclei) obtained by measuring at three evenly distributed points along CA3a/b. CA3 cells were counted in a single optical section to eliminate the issue of cell overlap and normalized to linear CA3 distance (in mm) to minimize the potential confounding effect of cell loss at the outer/inner aspect of the cell layer.

Mossy fiber sprouting was qualitatively evaluated (blindly) based on the ZnT3 staining pattern, using a modified scoring system [based roughly on the Timm scoring system of Tauck and Nadler (1985)] as follows: 0, no evidence for sprouting; 1, rare ZnT3+ stain in the GCL only; 2, some ZnT3+ in the GCL with rare ZnT3 in the IML; and 3, clear ZnT3+ puncta in IML. In no image from either genotype did there appear to be more than three putative ZnT3+ terminals in the GCL or IML within the region imaged. Ki67 staining was quantified from confocal stacks obtained using a 40× objective, and all Ki67+ nuclei within 10 µm of the SGZ were included in the analysis and normalized to GCL volume. Dcx staining was very dense in all mice of this age and quantified by measuring the average fluorescence intensity of Dcx+-stained tissue within the GCL, from a single 40× confocal section imaged near the surface of the slice, using identical side-by-side staining and imaging protocols. Astrocytes were quantified from 15 µm tall image stacks obtained from the granule cell and molecular layers using a 40× objective and defined as GFAP+ processes emanating from a clear central location (soma) that was within the imaged volume. Microglia were quantified from single confocal sections obtained at 40×, as cells with a clear soma present within the image based on Iba1 staining pattern. Due to low density of positive cells, Mac-2+ cells were quantified from confocal image stacks of the dentate, including the molecular layer, GCL, and hilus, acquired at 10× and normalized to dentate volume imaged.

### Statistics

The quantified data from each of the sections per experiment (as described above) were averaged to provide one data point for each measurement per animal (*n* = 1). A separate investigator unblinded these aggregate values and averaged them for each genotype, thus *n* is equal to the number of mice analyzed for each measurement. Data for each experiment are presented as ±standard error of the mean (SEM).

Statistical analyses were performed using Prism software (GraphPad), and statistical information for each comparison is presented in Table 2. For continuous numerical data (cell densities, layer width, fluorescence), normality of each dataset was first determined by the Shapiro–Wilk test. Group means were compared using two-tailed unpaired *t* tests for normally distributed data and the Mann–Whitney nonparametric test for non-normally distributed data. Significance was set at *p* < 0.05. Additional details on any methods, raw data, and processed data will be provided upon request.

**Table 2.** Statistical table

Comparison	Data structure	Statistical test	Difference between means	Power (95% confidence intervals)
Baseline c-*fos*+ total cell density	Non-normal distribution (*W* = 0.6411)	Mann–Whitney test	1.603 (medians)	Rank sums 56 vs 64
Baseline c-*fos*+ total cell density (including outlier)	Non-normal distribution (*W* = 0.4324)	Mann–Whitney test	1.964 (medians)	Rank sums 56 vs 80
Baseline c-*fos*+ semilunar cell density	Non-normal distribution (*W* = 0.6780)	Mann–Whitney test	19.14 (medians)	Rank sums 58 vs 62
Baseline c-*fos*+ granule cell density	Non-normal distribution (*W* = 0.6960)	Mann–Whitney test	1,649 (medians)	Rank sums 57 vs 63
Baseline c-*fos*+ total cell density: ventral	Normal distribution	Unpaired *t* test	315	−853 to 1,482
Baseline c-*fos*+ total cell density: ventral (including outlier)	Non-normal distribution (*W* = 0.5413)	Mann–Whitney test	643 (medians)	Rank sums 33 vs 45
Baseline *ΔFosB* GCL intensity	Non-normal distribution (*W* = 0.7748)	Mann–Whitney test	−83.13 (medians)	Rank sums 46 vs 45
Baseline *ΔFosB* GCL intensity (including outlier)	Non-normal distribution (*W* = 0.6061)	Mann–Whitney test	−76.98 (medians)	Rank sums 46 vs 59
Handling seizure score	Normal distribution	Unpaired *t* test	3.250	2.638 to 3.862
Handling c-*fos*+ cell density (WT vs KO)	Normal distribution	Unpaired *t* test	−3.834	−6.919 to 0.7493
Handling c-*fos*+ cell density: ventral	Normal distribution	Unpaired *t* test	−3,909	−7,169 to −650
Handling *ΔFosB* intensity	Normal distribution	Unpaired *t* test	468	167 to 768
WT: baseline vs handled c-*fos*+ cell density	Non-normal distribution (*W* = 0.6411)	Mann–Whitney test	−0.278 (medians)	Rank sums 54 vs 24
KO: baseline vs handled c-*fos*+ cell density	Normal distribution	Unpaired *t* test	−5.895	−9.456 to −2.334
PTZ seizure score	Non-normal distribution (*W* = 0.6068)	Mann–Whitney test	5.000 (medians)	Rank sums 37.5 vs 98.5
PTZ c-*fos*+ cell density	Non-normal distribution (*W* = 0.7440)	Mann–Whitney test	71,400 (medians)	Rank sums 47 vs 89
PTZ *c-fos*+ cell density: ventral	Non-normal distribution (*W* = 0.7572)	Mann–Whitney test	57,100 (medians)	Rank sums 47 vs 89
PTZ *ΔFosB* GCL intensity	Normal distribution	Unpaired *t* test	455	−11 to 921
PTZ high c-*fos* vs low c-*fos*	Normal distribution	Unpaired *t* test	3.000	−12.6 to 18.6
Calretinin+ cell density	Normal distribution	Unpaired *t* test	107.3	7.566 to 207.0
Hilar cross-sectional area	Normal distribution	Unpaired *t* test	0.005	−0.015 to 0.005
GCL cross-sectional area	Normal distribution	Unpaired *t* test	−0.011	−0.041 to 0.018
CA3 pyramidal cell layer thickness	Non-normal distribution (*W* = 0.575)	Mann–Whitney test	5.75 (medians)	Rank sums 38 vs 40
CA3 pyramidal cell density	Normal distribution	Unpaired *t* test	47.00	−88.44 to 182.4
Mossy fiber sprouting	Non-normal distribution (*W* = 0.7013)	Mann–Whitney test	−0.375 (medians)	Rank sums 51.5 vs 26.5
Ki67+ cell density	Normal distribution	Unpaired *t* test	13.61	−3.455 to 30.67
DCX intensity	Normal distribution	Unpaired *t* test	−120.0	−585.3 to 345.4
PV+ cell density in the GCL	Normal distribution	Unpaired *t* test	356.3	−348.6 to 1,061
PV+ cell density in the hilus	Normal distribution	Unpaired *t* test	4.014	−196.5 to 204.6
PV+ cell density in CA3	Normal distribution	Unpaired *t* test	3.285	−4.953 to 11.52
GFAP+ cell density	Normal distribution	Unpaired *t* test	2.460	−2.207 to 7.126
GFAP intensity	Normal distribution	Unpaired *t* test	8.395	−38.25 to 55.04
Iba1 cell density	Normal distribution	Unpaired *t* test	2.630	−36.21 to 41.47
Mac-2 cell density	Normal distribution	Unpaired *t* test	524	−178 to 1,225

## Results

### Juvenile *CACNA2D2* KO mice do not have increased granule cell activity at baseline

*CACNA2D2* mutant mice begin to experience spontaneous generalized convulsions at approximately weaning age (3–4 weeks), which manifest as excited jumping/falling, reeling to one side, stiffening of hindlimbs and tail, and periodic paraplegia (Meier, 1968). To assess whether this phenotype was associated with elevated neuronal activity in the hippocampus, we stained the hippocampal dentate gyrus harvested from a 1-month-old mice for the activity-dependent immediate early gene c-*fos*. Transcription and translation of c-*fos* are transiently increased after neuronal firing, and thus anti-c-*fos* staining serves as a reliable histochemical marker of recent neuronal activity (Morgan et al., 1987; Bullitt, 1990). We focused on the dentate gyrus, which serves as the gateway for electrical activity to enter the hippocampus and has a very sparse baseline activity in healthy mice (Jung and McNaughton, 1993).

In the dentate, both WT and *CACNA2D2* KO mice had similarly sparse densities of c-*fos*+ cells within and immediately adjacent to the GCL (Fig. 1*A*,*B*). Although not quantified explicitly, the c-*fos*+ cells were anecdotally greater in the suprapyramidal blade of the dentate (closer to CA1) than those in the infrapyramidal blade in both genotypes (Fig. 1*A*). Many c-*fos*+ cells were also found along the border of the GCL and IML, the typical location for semilunar granule cells (Williams et al., 2007). To examine whether these cells might have a selective activation pattern, we separately categorized the c-*fos*+ cells within one cell body diameter of the GCL–IML border as putative semilunar cells and the c-*fos*+ cells fully within the GCL as putative granule cells. c-*fos*+ cell density was not different between genotypes in either location (putative semilunar cells: WT = 862 ± 133 cells/mm^3^, KO = 1,185 ± 325 cells/mm^3^, *n* = 8, 7 mice, *p* = 0.54; putative granule cells: WT = 5,350 ± 530 cells/mm^3^, KO = 6,250 ± 810 cells/mm^3^, *n* = 8, 7 mice, *p* = 0.46). These data suggest that at baseline, there is no difference in the hippocampal dentate gyrus activity in *CACNA2D2* KO mice.

In the above analyses, a single KO mouse was excluded from the group data, as its c-*fos*+ cell density was over 100 standard deviations above the mean value for the rest of the group, with essentially every single dentate granule cell staining as c-*fos*+ (Fig. 1*C*). Based on this expression pattern, this mouse almost definitely had a seizure which had propagated through the dentate gyrus in the hour prior to fixation (Morgan et al., 1987; Barone et al., 1993). We felt it was appropriate to exclude it from the group mean data, as it likely did not represent a resting baseline c-*fos* expression pattern. However, even if this dramatic outlier was included in the analysis, there still was no statistically significant difference between the two genotypes in the basal level of granule cell activation, as measured by c-*fos* expression (total c-*fos*+ cell density with outlier included: WT = 6,210 ± 610 cells/mm^3^; KO = 68,390 ± 60,960 cells/mm^3^; *n* = 8, 8 mice; *p* = 0.23). Thus, *CACNA2D2* KO mice do not appear to have grossly increased hippocampal dentate gyrus activity when resting in their home cages.

The dorsal and ventral regions of the hippocampus have been differentially implicated in epilepsy-related circuit activity (Toyoda et al., 2013; Buckmaster et al., 2022). To more broadly assess c-*fos* expression in the dentate gyrus in *CACNA2D2* KO mice, we stained the ventrolateral sections of the hippocampus (AP −3.3 mm from bregma) for c-*fos*. Similar to the findings in more dorsal sections, there was no difference in c-*fos* expression in more ventral hippocampus between WT and KO mice (total c-*fos*+ cell density with outlier excluded: WT = 2,090 ± 306 cells/mm^3^, KO = 2,405 ± 432 cells/mm^3^, *n* = 6, 5 mice as two *n* from each group were missing ventral samples, *p* = 0.56; total c-fos+ cell density with outlier included: WT = 2,090 ± 306 cells/mm^3^, KO = 9,977 ± 7,580 cells/mm^3^, *n* = 6, 6 *p* = 0.39).

Another member of the Fos family of immediate early genes, FosB, is similarly expressed in an activity-dependent manner, and in particular, a splice variant of the FosB gene (ΔFosB) is commonly used as a marker of chronically elevated neuronal activity due to its significantly extended protein half-life (Stephens et al., 2020). We stained sections from the WT and *CACNA2D2* KO mice above for *ΔFosB* to evaluate whether KO mice have more chronically elevated levels of activity. There was no difference in *ΔFosB* expression between WT and KO mice (Fig. 1*E*), again with either the outlier mouse excluded or included in the analysis (*ΔFosB* staining intensity with outlier excluded: WT = 432 ± 82 arbitrary units (AU), KO = 481 ± 105 AU, *n* = 7, 6 mice, *p* = 0.73; c-*fos*+ cell density with outlier included: WT = 481 ± 105 AU, KO = 947 ± 474 AU, *n* = 7, 7 mice, *p* = 0.46).

### Altered dentate granule cell activity after induced seizures in *CACNA2D2* KO mice

*CACNA2D2* mutant mice can experience convulsive behavioral seizures after gentle handling (Meier, 1968), and *CACNA2D2* KO mice have been observed to have convulsions in our animal facility with cage transfers. Thus, we considered the possibility that the KO mouse with widespread c-*fos* activation (Fig. 1*C*) may have experienced a seizure involving the dentate gyrus due to handling shortly before killing for tissue harvest. In the experiment above, mice were typically killed in <15 min after leaving their home cages in the animal facility. Thus, the majority of mice may not have had ample time to express detectable c-*fos* after possible handling-induced convulsions, since c-*fos* takes ∼60 min to achieve peak expression after activation (Morgan et al., 1987). To explicitly assess a potential relationship between handling and hippocampal activation, we killed WT and KO mice exactly 1 h after gentle handling to determine whether handling impacts hippocampal granule cell activity.

Mice were picked up by the base of their tails, observed for 5 s, gently scruffed by the nuchal fur, and then placed in an observation cage, in a manner similar to our typical cage transfers prior to killing, with the only difference being continuous observation for 1 h prior to killing. Although WT mice immediately resumed typical resting and exploratory behaviors after handling, *CACNA2D2* KO mice exhibited seizure behavior similar to prior descriptions of handling-induced convulsions in spontaneous *CACNA2D2* mutant (*ducky*) mice (Snell, 1955; Meier, 1968), in which mice would at times rear and fall over, with bilateral limb clonus, typically beginning 1–2 min after being placed in the cage and persisting for the remainder of the observation. In some mice, the convulsive behaviors would intermittently pause for minutes at a time, during which the mice appeared to be resting quietly, followed by reappearance of similar convulsions. This behavior appeared distinct from the continuous stage 1/2-type seizures characteristic of pilocarpine-induced status epilepticus (Shibley and Smith, 2002) as there did not appear to be any convulsive behavior, or even tremor, during resting periods after handling.

**Figure 2.**
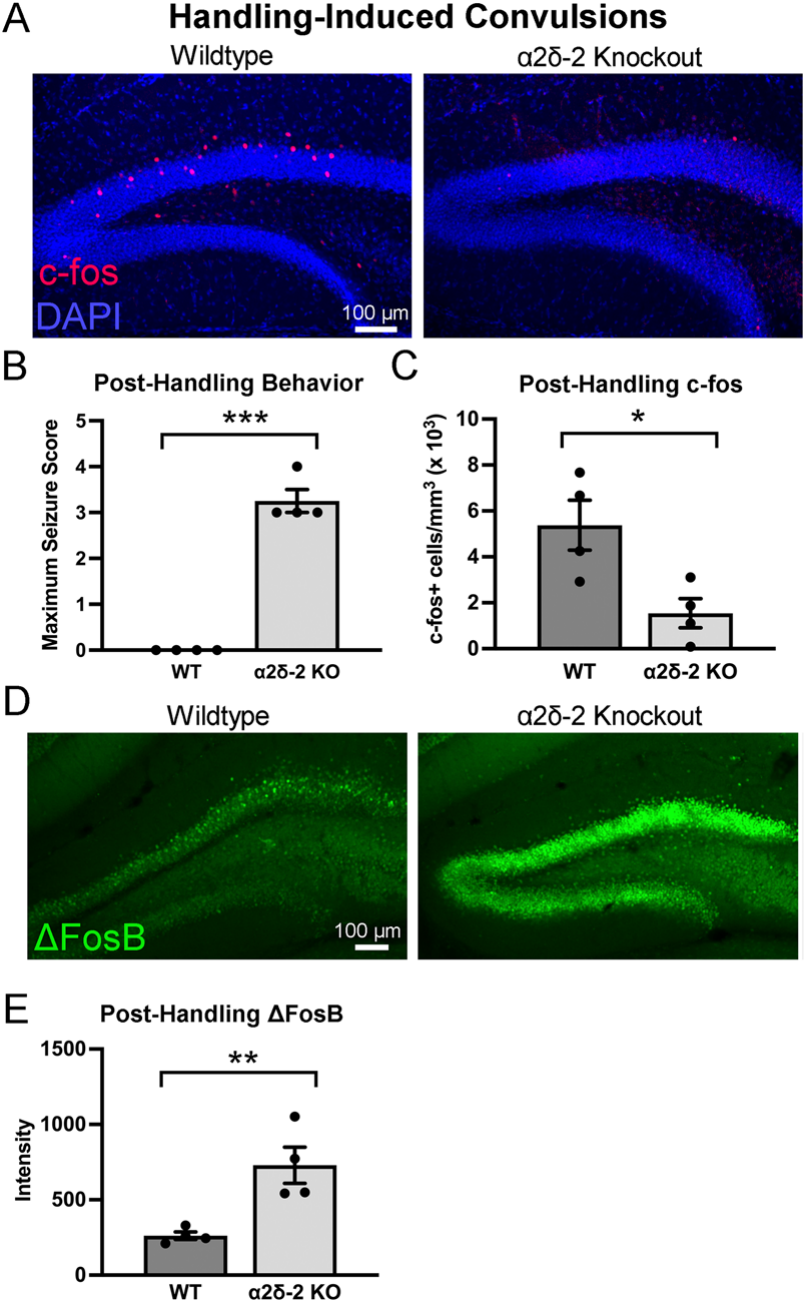
Granule cell activation after handling-induced convulsions. ***A***, Images of c-*fos* expression (red) 1 h after gentle handling in representative WT and KO mice. Cell nuclei were visualized using DAPI stain (blue). ***B***, Gentle handling caused convulsive seizures in *CACNA2D2* KO, but not WT, mice (*n* = 4, 4; ***p < 0.0001). ***C***, Granule cell c-*fos* expression was reduced in *CACNA2D2* mice after handling-induced convulsions (*n* = 4, 4; *p = 0.023). ***D***, Images of *ΔFosB* expression (green) 1 h after gentle handling in representative WT and KO mice. ***E***, Granule cell *ΔFosB* expression was increased in *CACNA2D2* mice after handling-induced convulsions (*n* = 4, 4; **p = 0.009).

One hour after gentle handling, WT mice had dentate c-*fos*+ expression patterns very similar to WT mice killed in our initial control experiments above (Figs. 1*A*, 2*A*; total c-*fos*+ cell density: WT immediate harvest = 6,210 ± 610 cells/mm^3^, WT handled and delayed harvest = 5,380 ± 1,090 cells/mm^3^, *n* = 8, 4 mice, *p* = 0.81). Surprisingly, however, despite clear convulsive activity (Fig. 2*B*), handled *CACNA2D2* KO mice actually demonstrated reduced dentate c-*fos* expression 1 h after handling when compared with WT littermates (total c-*fos*+ cell density: WT handled and delayed harvest = 5,380 ± 1,090 cells/mm^3^, KO handled and delayed harvest = 1,540 ± 640 cells/mm^3^, *n* = 4, 4 mice, *p* = 0.023; Fig. 2*A–C*). KO mice that were handled also had significantly fewer c-*fos*+ cells than KO mice which were immediately harvested (*n* = 8, 4; *p* < 0.005), suggesting that the decreased granule cell activity was a consequence of handling. This decreased c-*fos* expression in handled KO mice was also evident in the ventral sections (WT handled = 5,535 ± 1,131 cells/mm^3^; KO handled = 1,626 ± 704 cells/mm^3^; *n* = 4, 4 mice; *p* = 0.026). Thus, not only was posthandling convulsive activity not associated with increased hippocampal c-*fos* expression 1 h after handling, c-*fos* expression was actually below basal levels.

Although reduced c-*fos* expression typically indicates reduced neuronal activation, one alternative possibility is that seizure-associated immediate early gene expression follows different dynamics in KO mice. c-*fos* expression can be potently inhibited by a different activity-dependent gene, Δ*FosB*, which typically expresses with a slightly delayed time course in WT mice after cellular activity, and thus could contribute to postictal suppression of c-*fos* expression (Peng and Houser, 2005; Lamothe-Molina et al., 2022). Given the resemblance of the minimal c-*fos* expression in posthandling KO mouse tissue to the appearance of postictal hippocampi (Morgan et al., 1987), we wondered whether KO mice might also have changes in *ΔFosB* expression after handling. When we stained the hippocampal tissue from the same cohort of WT and KO mice (after handling) above, we surprisingly found dramatically increased *ΔFosB* staining in KO mice compared with that in WT mice (Fig. 2*D*,*E*). As *CACNA2D2* KO mice do not have elevated *ΔFosB* staining at baseline, together these data suggest that handling-induced convulsions actually are associated with substantial granule cell activation but that Δ*FosB* expression, which is normally somewhat delayed after seizures, is induced quickly. Although we are not able to conclude that the increase in the *ΔFosB* expression caused the downregulation in c-*fos* in our KO samples, prior investigations demonstrate that *ΔFosB* suppresses c-*fos* in the dentate (Lamothe-Molina et al., 2022), which is one putative mechanism that might be engaged here.

In addition to spontaneous and handling-induced behavioral seizures, *CACNA2D2* KO mice have a markedly decreased seizure threshold in response to low doses of the convulsant PTZ (Ivanov et al., 2004). As behavioral seizures resulting from gentle handling were paradoxically associated with decreased c-*fos* activity in the dentate gyrus (Fig. 2), we examined whether low-dose PTZ-induced seizures might also be associated with altered dentate granule cell activity in KO mice. All mice received a subthreshold PTZ dose (30 mg/kg, i.p.), which typically does not cause seizures in WT mice but causes seizure-like activity in a majority of *CACNA2D2* KO mice (Ivanov et al., 2004).

**Figure 3.**
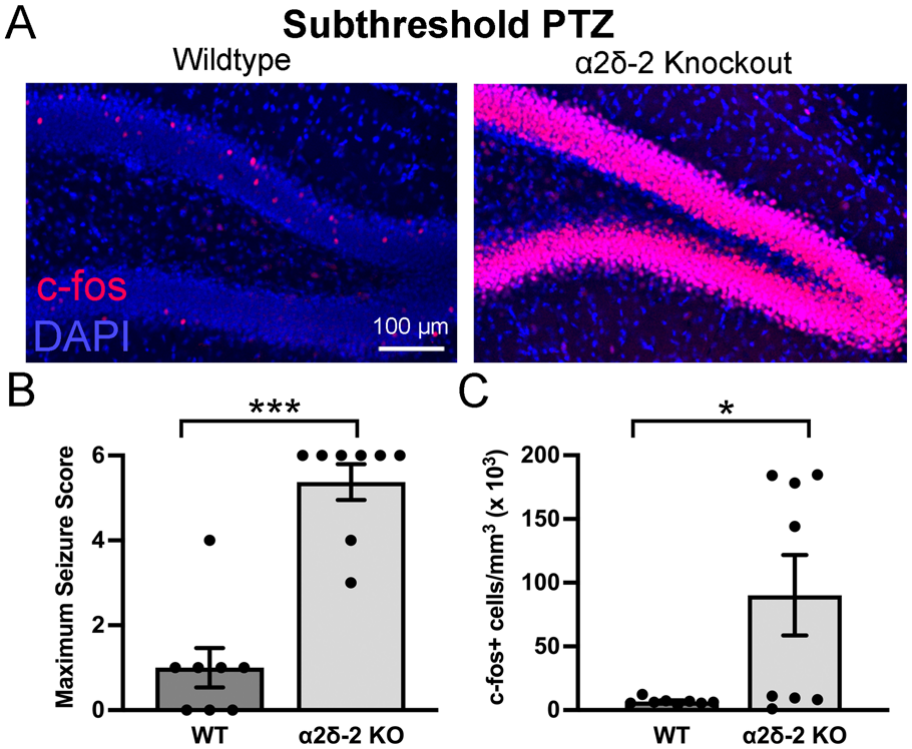
*CACNA2D2* KO mice have a reduced threshold for PTZ -induced granule cell activation. ***A***, Dentate c-*fos* expression (red) after low-dose (30 mg/kg) PTZ administration in WT and KO mice. ***B***, Maximum seizure intensity after PTZ (*n* = 8, 8; ***p = 0.005). ***C***, c-*fos*+ cell density after PTZ (*n* = 8, 8; *p = 0.028).

As previously reported (Ivanov et al., 2004), while WT mice displayed little to no behavioral response to the subthreshold PTZ dose, *CACNA2D2* KO mice consistently responded to this dose with convulsive seizure activity (Fig. 3*A*,*B*; WT: one of eight mice with seizure score ≥3; KO: eight of eight mice with seizure score ≥3; *p* = 0.005). Unlike handling-induced convulsions, which persisted at a lower and intermittent grade throughout the 1 h observation period, PTZ-induced seizures typically began ∼5 min after injection in KO mice and spontaneously resolved in under 10 min. Unlike KO mice with handling-induced convulsions, KO mice had significantly increased c-*fos* activation in the dentate gyrus after PTZ (Fig. 3*A*,*C*). This increase was also noted in separately stained samples from more ventral dentate gyrus (WT post-PTZ = 5,702 ± 561 c-fos+ cells/mm^3^; KO post-PTZ = 54,872 ± 15,695 c-fos+ cells/mm^3^; *n* = 8, 8 mice; *p* = 0.028). Ventral c-*fos* expression in individual KO mice after PTZ was highly correlated with dorsal c-*fos* expression based on a simple linear regression (*r*^2^ = 0.75; slope, 0.101; *F* = 18.1; *p* = 0.005), suggesting that the c-*fos* expression patterns after PTZ extended throughout the hippocampus. In apparent contrast with the reduced c-*fos* expression after handling, these data suggest that the seizures induced by PTZ are accompanied by increased functional hippocampal network recruitment as measured by increased c-*fos* expression.

This cohort of KO mice expressed a bimodal distribution of c-*fos* activation, in which half (four of eight) of the KO mice had clearly detectable c-*fos* expression in nearly every granule cell, while three of the remaining four mice had c-*fos*+ cell densities near, but still above, the mean for WT mice after PTZ. To more closely examine a potential relationship between seizure intensity and c-*fos* labeling, we added the maximum recorded seizure score for each minute after PTZ for each mouse, to give an “integrated” seizure score for each KO mouse over the first 15 min (after which seizure activity had largely subsided). There was no difference in seizure intensity between “high-c-*fos*” and “low-c-*fos*” KO mice (“high c-*fos*” KO average integrated seizure score = 38.5 ± 3.8; “low c-fos” KO = 41.5 ± 5.2; *n* = 4; 4, *p* = 0.66). Thus, somewhat to our surprise, there was no clear association between the denser c-*fos* expression and post-PTZ seizure scores. Another possibility is that a recent seizure (<24 h prior to PTZ) may have suppressed subsequent c-*fos* activation in the “low c-*fos*” mice (Peng and Houser, 2005). Unfortunately, as mice were not closely observed during this time interval, it is not known which, if any, KO mice had seizures during this interval prior to testing.

As an alternative measure of granule cell activation by subthreshold PTZ, we stained the tissue from WT and KO mice for the more delayed activity-dependent gene, *ΔFosB*. Although there was a trend toward increased *ΔFosB* expression after PTZ in KO mice, it did not reach statistical significance (Δ*FosB* staining intensity: WT = 370 ± 71 AU; KO = 825 ± 237 AU; *n* = 8, 8 mice; *p* = 0.055). Interestingly, Δ*FosB* staining intensity also had a bimodal distribution in KO mice, but without an obvious relationship to c-*fos* staining density. There were four mice in the PTZ group that had a substantial increase in *ΔFosB* staining intensity (∼4-fold above the WT mean) and four (of the eight) mice that were fairly similar to WT controls. However, these four “high *ΔFosB*” mice were not the same as the four “high c-*fos*” mice. Assessed via a simple linear regression, the relationship between c-*fos*+ cell density and *ΔFosB* expression in each KO mouse was not correlated or anticorrelated (*r*^2^ = 0.11; slope, 0.002; *F* = 0.73; *p* = 0.42).

### Increased hilar mossy cell density without CA3 cell loss in *CACNA2D2* KO mice

Human TLE is characterized by numerous histopathological changes in the hippocampus, primarily within the dentate gyrus, many of which are recapitulated in both chemoconvulsant and genetic mouse models of epilepsy (Loscher, 2017). Hippocampal activation during seizures in *CACNA2D2* KO mice was clearly evidenced by increased c-*fos* expression within the dentate GCL after subthreshold PTZ administration (Fig. 3) and increased dentate *ΔFosB* after handling-evoked convulsions (Fig. 2). It was also observed on at least one occasion in an otherwise seemingly unprovoked manner (Fig. 1*C*). Thus, we considered the possibility that functional and structural changes in the hippocampus might exist in these mice and potentially contribute to dentate hyperexcitability. We examined whether *CACNA2D2* KO mice demonstrated histopathological changes in the hippocampus similar to those frequently observed in mouse models of TLE (Sharma et al., 2007), beginning with the loss of excitatory cell populations in the dentate hilus and CA3 pyramidal cell layer.

**Figure 4.**
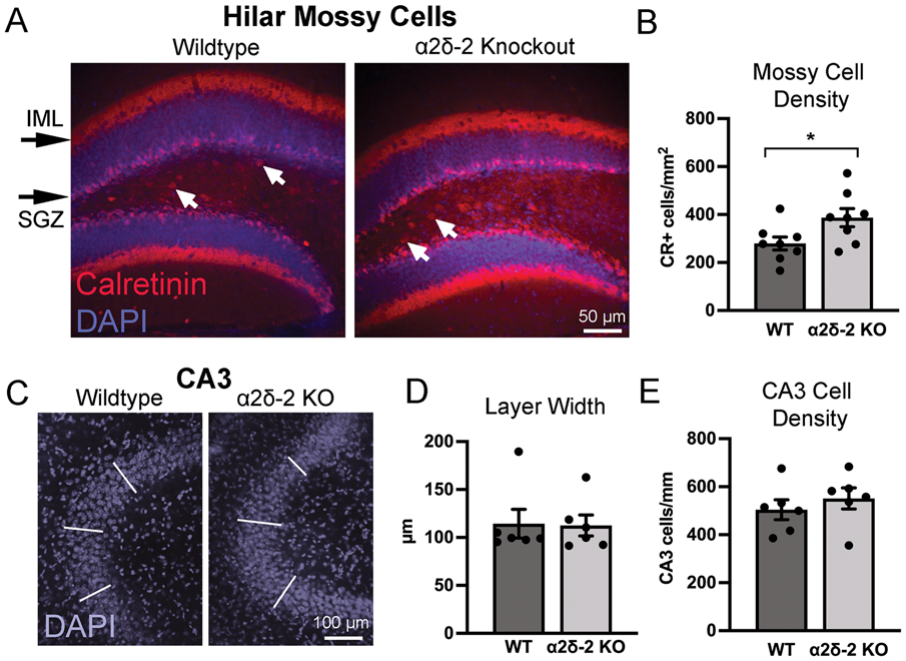
Hilar mossy cell and CA3 cell densities in *CACNA2D2* KO mice. ***A***, Hilar mossy cells were identified as calretinin-positive cells (red; arrows) located within the hilar space and distinct from the small calretinin-positive immature granule cells in the SGZ. Mossy cell axons appear as a calretinin-stained band in the IML, just outside of the GCL (blue, DAPI). ***B***, Quantification of hilar mossy cell density (*n* = 8, 8; *p = 0.037). ***C***, CA3 pyramidal cell layer seen in cross section, visualized using DAPI nuclear stain (blue). White lines designate three separate locations at which CA3 pyramidal layer width was measured. ***D***, Quantification of CA3 pyramidal cell layer width (*n* = 6, 6; *p* = 0.94). ***E***, Quantification of CA3 pyramidal cell layer density (*n* = 6, 6; *p* = 0.46).

The density of glutamatergic hilar mossy cells was quantified by staining for the calcium-buffering protein calretinin, a selective marker for excitatory mossy cells within the dentate hilus (Blasco-Ibanez and Freund, 1997). As calretinin (CR) is also expressed by immature granule cells ∼1–2 weeks after mitosis (Brandt et al., 2003), we excluded small CR+ somata in the SGZ that were most likely immature granule cell neurons, and only counted large CR+ cells within the hilar region. Although hilar mossy cell loss is a prominent characteristic of many mouse models of epilepsy (Scharfman, 2016), we found that not only was the mossy cell population intact within the dentate hilus of *CACNA2D2* KO mice, but there was a small statistically significant increase in mossy cell density in KO mice (Fig. 4*A*,*B*). This was not associated with a statistically significant difference in the hilar cross-sectional area between WT and KO mice (WT = 0.068 ± 0.008 mm^2^; KO = 0.063 ± 0.004 mm^2^; *p* = 0.32) or the cross-sectional GCL area (WT = 0.095 ± 0.011 mm^2^; KO = 0.084 ± 0.008 mm^2^; *p* = 0.42), indicating no gross anatomical distortion or shrinkage in KO mice. However, the slight nonsignificant shrinkage in either the hilar space or GCL area might otherwise magnify the increase in hilar mossy cell density, if similar numbers of mossy cells per hilus were being “squeezed” into a slightly smaller space. Regardless, it is at least clear that mossy cell density was not reduced in KO mice at the age of spontaneous behavioral seizure onset.

Similarly, degeneration of hippocampal CA3 pyramidal cells occurs in numerous mouse models of epilepsy as well as in pathological specimens from human patients, which over time can contribute to an atrophied, sclerotic hippocampus (de Lanerolle et al., 2012). To assess CA3 cell loss, we quantified both CA3 pyramidal cell layer width and CA3 pyramidal cell density by quantifying DAPI-stained nuclei in the hippocampal sections. Neither of these metrics was different between *CACNA2D2* WT and KO mice (Fig. 4*C–E*), indicating that CA3 pyramidal cell loss is not a characteristic of *CACNA2D2* KO mice at this age.

### Juvenile *CACNA2D2* KO mice do not exhibit retrograde mossy fiber axon sprouting

One pathognomonic histopathological hallmark of TLE involves the retrograde sprouting of granule cell axons, also known as the mossy fibers (Nadler, 2003). Although the mechanisms driving mossy fiber sprouting are unclear, these fibers form recurrent excitatory circuits and may contribute to the initiation or propagation of seizures (Tauck and Nadler, 1985; Dudek and Shao, 2004; Hendricks et al., 2019).

**Figure 5.**
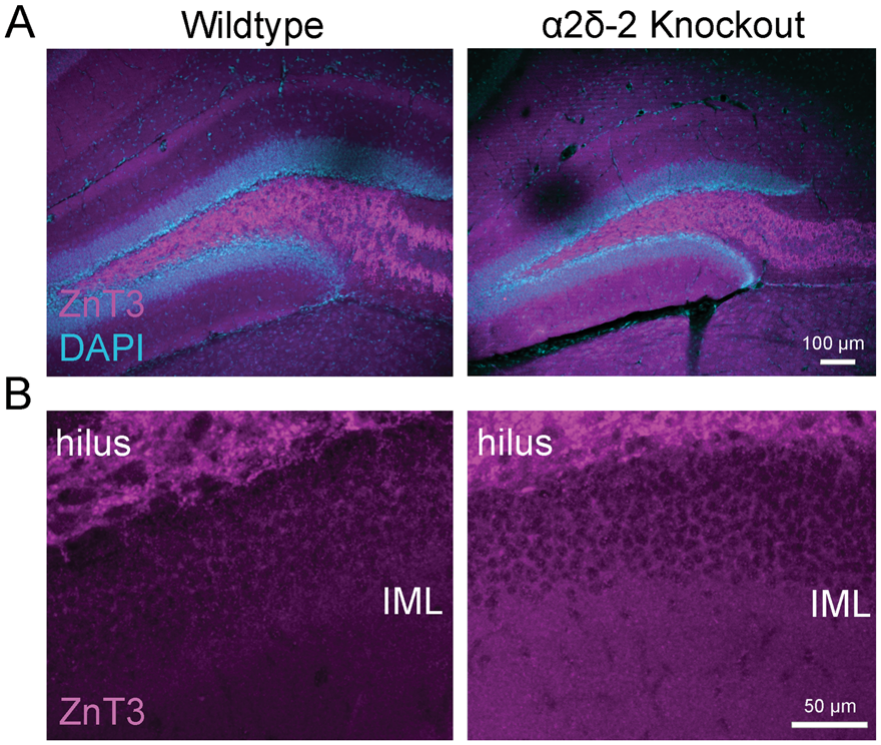
Mossy fiber sprouting is absent in *CACNA2D2* KO mice. ***A***, Granule cell mossy fiber axon terminals in WT and KO mice were identified by staining for the mossy fiber terminal marker, ZnT3 (magenta). GCL highlighted with DAPI (blue). ***B***, ZnT3-stained terminals were exclusively found within the hilar space, and not within the IML, which would have indicated pathological retrograde sprouting.

To assess whether juvenile *CACNA2D2* KO mice manifest mossy fiber sprouting, we stained the hippocampal sections for Zinc Transporter 3 (ZnT3) protein, which is highly expressed in mossy fiber terminals in both healthy and epileptic tissues (Murphy et al., 2011). In WT animals, ZnT3 was detected as dense staining throughout the dentate hilus and among the proximal dendrites of CA3 pyramidal cells (stratum lucidum), consistent with the known locations of mossy fiber synapses in healthy mice. KO mice had the same distribution of ZnT3-stained terminals (Fig. 5) and, most notably, a lack of ZnT3-stained puncta within the dentate IML, where spouted mossy fiber terminals are found in the epileptic tissue (Murphy et al., 2011). No sections from any mouse had consistently observed ZnT3-positive boutons localized within the GCL or IML across sections (Fig. 5), and a blinded quantification based on a modified Timm's scoring system for mossy fiber sprouting showed no difference between groups (modified Timm's score: WT = 0.52 ± 0.19; KO = 0.13 ± 0.09; *n* = 6 per group; *p* > 0.05).

### Granule cell proliferation and hippocampal neurogenesis are not increased in juvenile *CACNA2D2* mutants

The hippocampal dentate gyrus is characterized by ongoing granule cell neurogenesis in adulthood, and the rate of hippocampal adult neurogenesis is modulated by numerous biological contingencies, including neurological disease (Gage, 2002; Eisch et al., 2008). Seizures dramatically affect hippocampal neurogenesis in the dentate, causing increased neural stem cell proliferation, as well as accelerated dendritic outgrowth, outward cell body migration, and accelerated synaptic innervation of newly generated neurons (Parent et al., 1997; Overstreet-Wadiche et al., 2006). The strong association of seizures with aberrant neurogenesis has led to the suggestion that aberrant neurogenesis might directly contribute to the pathogenesis of epilepsy (Danzer, 2012; Parent and Kron, 2012; Cho et al., 2015).

**Figure 6.**
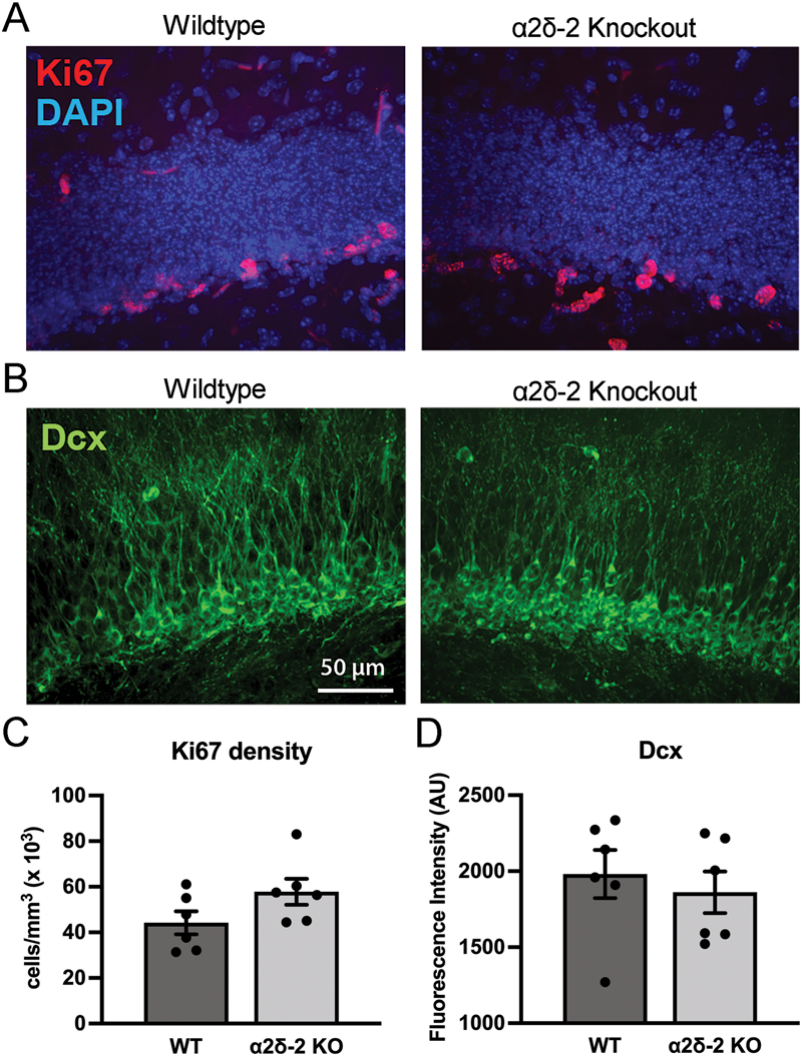
Hippocampal cell proliferation and granule cell neurogenesis are unchanged in juvenile *CACNA2D2* KO mice. ***A***, Mitotic cells in the dentate SGZ identified by the proliferation-associated nuclear marker Ki67 (red) in WT and KO mice; GCL nuclei were counterstained with DAPI (blue). ***B***, Immature granule cell neurons identified by expression of the microtubule-associated protein Dcx (green) in WT and KO dentate sections. ***C***, Quantification of Ki67+ cell density (*n* = 6, 6; *p* = 0.11). ***D***, Quantification of Dcx+ cell density using staining fluorescence intensity in the GCL (*n* = 6, 6; *p* = 0.58).

To quantitatively assess hippocampal granule cell neurogenesis in juvenile *CACNA2D2* KO mice, we first stained the hippocampal sections for the nuclear marker, Ki67. Ki67 is expressed by cells undergoing active mitosis (Scholzen and Gerdes, 2000) and commonly used as a measure of cell proliferation. In hippocampal sections from both WT and KO mice, Ki67+ nuclei were found within/near the dentate SGZ, as would be expected for proliferating neural stem cells from which granule cells are generated, which reside in this region (Fig. 6*A*). There was no difference in the density of the Ki67+-labeled cells between groups (Fig. 6*C*), indicating that *CACNA2D2* KO does not alter cell proliferation within the neurogenic niche of the dentate gyrus.

As Ki67 is not selective for neurogenic precursor cells, we stained the hippocampal sections for Dcx, a microtubule-associated protein that is expressed by immature granule cell neurons early after mitosis. Again, both genotypes had similarly dense appearing Dcx+ staining (Fig. 6*B*), indicative of the robust neurogenesis that is typical of juvenile mice at this age. Quantification of Dcx fluorescence demonstrated no significant difference between groups (Fig. 6*D*), suggesting no difference in the density of immature, recently born granule cells in KO mice. Together with a lack of difference in cell proliferation, these data indicate that *CACNA2D2* KO mice do not have gross abnormalities in dentate granule cell neurogenesis at this age.

### Parvalbumin-positive interneuron density is unchanged in *CACNA2D2* KO mice

α2δ-2 is highly expressed in PV+ interneurons (Cole et al., 2005), which mediate feedforward inhibition and control neuronal excitability in the hippocampus. In some models of epilepsy, PV+ interneuron density in the hippocampus is reduced, leading to the suggestion that deficits in inhibitory circuits might contribute to neuronal circuit hyperexcitability (Dudek and Sutula, 2007). Thus, to assess whether loss of the α2δ-2 protein is associated with a loss of PV+ interneurons, we stained the hippocampal sections from WT and *CACNA2D2* KO mice for PV.

**Figure 7.**
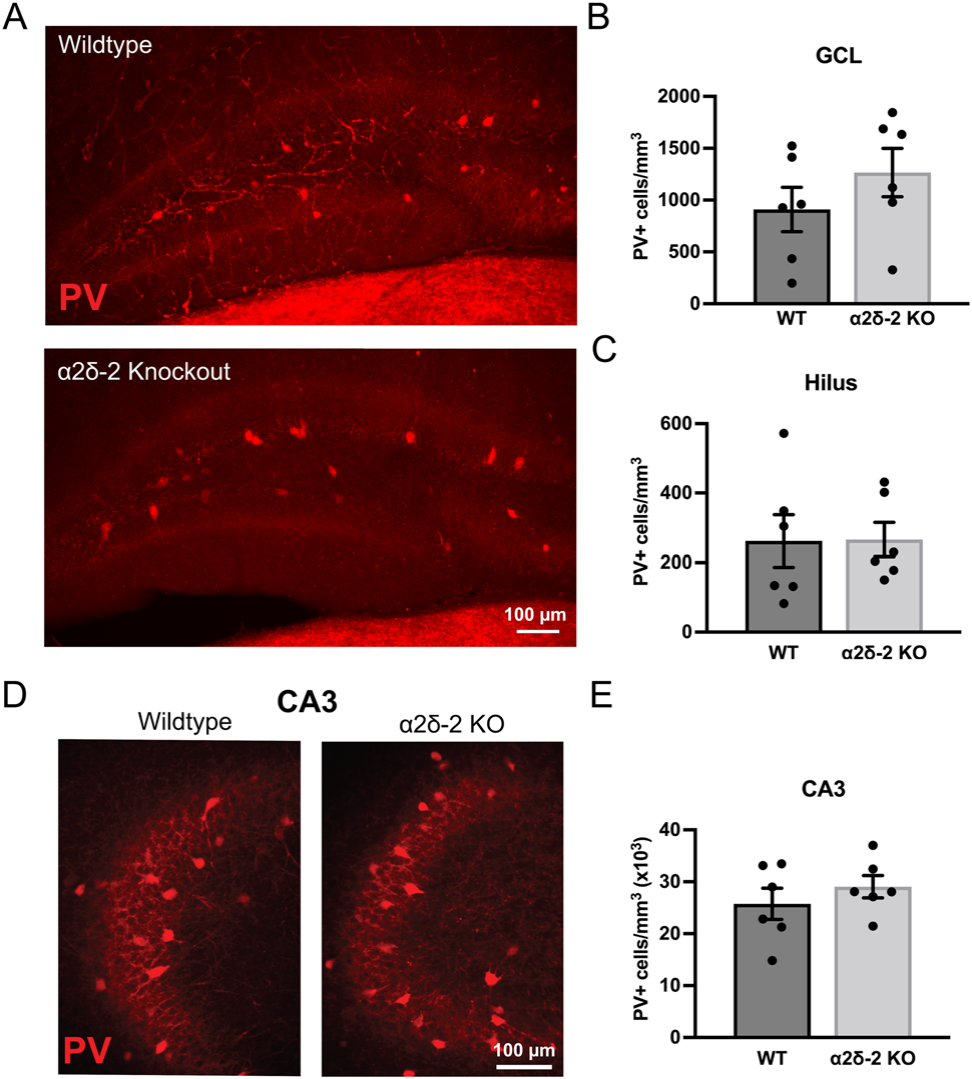
Parvalbumin interneuron staining in WT and *CACNA2D2* KO mice. ***A***, Confocal image projections of parvalbumin-stained (PV, red) dentate gyrus tissue from WT and KO mice. ***B***, ***C***, Quantification of PV+ cell density within both the GCL (***B***; *n* = 6, 6; *p* = 0.29) and dentate hilus (***C***; *n* = 6, 6; *p* = 0.97). ***D***, Confocal image projections of PV-stained hippocampal CA3 pyramidal cell layers from WT and KO mice. ***E***, Quantification of PV+ cell density in the CA3 pyramidal cell layer (*n* = 6, 6; *p* = 0.40).

In the dentate gyrus, PV+ cells were sparse and predominantly localized along the SGZ, the typical location of PV+ basket-type interneurons, as well as more rarely within the hilus and molecular layer. To focus on the basket-type PV cells most closely associated with feedforward inhibition in the dentate gyrus, we separately quantified PV+ cells within the GCL and dentate hilus. The PV+ cell density in both regions was not different between WT and KO mice (Fig. 7*A–C*).

As a change in PV+ cell density in CA3 could profoundly alter hippocampal circuit excitability, we also quantified PV+ cell density within the CA3 pyramidal cell layer. Again, there was no difference in the PV+ cell density within the CA3 pyramidal cell layer between WT and KO mice (Fig. 7*D*,*E*). Additionally, subjective assessments of cell morphology performed on WT and KO images after unblinding did not reveal any obvious difference in cell structure or axonal projections of PV+ neurons. Together, these data indicate that *CACNA2D2* KO is not associated with gross structural changes within the hippocampal PV+ interneuron cell network.

### Glial marker staining is not increased in *CACNA2D2* KO mice

Epilepsy is associated with glial activation (Dossi et al., 2018). Thus, neuronal hyperexcitability can occur as a direct consequence of neuronal injury or alternatively may be due to underlying increases in neuroinflammation that could secondarily contribute to epileptogenesis (Wetherington et al., 2008). Both astrocytes and microglia modulate neural circuit function and structure through both direct interactions with neurons and synapses and indirectly through the release of neuromodulatory and neurotropic factors (Christopherson et al., 2005; Ekdahl et al., 2009; Schafer et al., 2013). Thus, we immunohistochemically evaluated two hippocampal glial cell populations, astrocytes and microglia, in brain tissue from WT and *CACNA2D2* KO mice.

**Figure 8.**
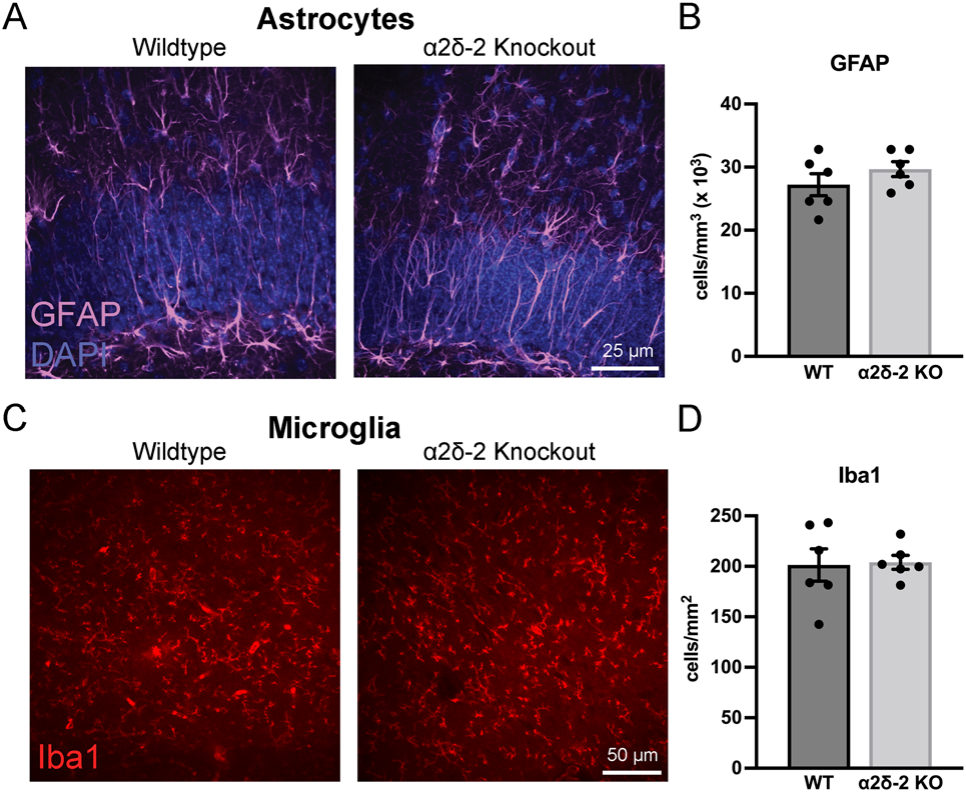
Glial activation is not changed in *CACNA2D2* KO mice. ***A***, Confocal stack image projections of GFAP-stained dentate GCLs (DAPI, blue) from WT and KO mice. ***B***, Quantification of GFAP+ cell densities (*n* = 6, 6; *p* = 0.29). ***C***, Confocal image of Iba1-stained (red) dentate molecular layer tissue from WT and KO mice. ***D***, Quantification of Iba1+ cell densities (*n* = 6, 6; *p* = 0.88).

To determine whether hippocampal astrocytes are altered in number or activation state in the absence of the α2δ-2 protein, we stained the hippocampal sections for GFAP, which both identifies astrocytes and increases in expression with astrocytic activation (Schiffer et al., 1986). GFAP+ cells with typical astrocytic spider-like morphology were found throughout the dentate molecular, granule cell, and hilar regions in both WT and KO mice and also included GFAP+ radial processes within the GCL, which resembled previously described radial glial stem cells. Quantification of the density of GFAP+ cells between WT and KO mice revealed no difference in the density of astrocytes between WT and KO mice (Fig. 8*A*,*B*). Additionally, as GFAP expression can increase in activated astrocytes, we compared immunohistochemical signal intensities between groups and also found no differences between them (WT = 359 ± 14 AU; 367 ± 16 AU; *n* = 6, 6; *p* = 0.70).

As microglia powerfully modulate neuronal circuits and play a role in secondary neuronal damage, we stained the hippocampal tissue for the microglial marker, Iba1. There was no difference in the density or distribution of microglia (Iba1) between the WT and KO mice (Fig. 8*C*,*D*). Similarly, there was no difference in the density of activated microglia, defined as those staining for the marker Mac-2 (Galectin-3), which was similarly low in both WT and KO mice (Mac-2+ cells in WT = 698 ± 206 cells/mm^3^; KO = 1,221 ± 238 cells/mm^3^; *n* = 6, 6 mice, *p* = 0.13; on average <3 cells per dentate GCL confocal stack). Together with the lack of change in GFAP immunohistochemistry, these data indicate that loss of the α2δ-2 protein is not associated with pathophysiological hippocampal glial cell proliferation or activation within the dentate.

## Discussion

Mice lacking the α2δ-2 protein have increased behavioral seizure susceptibility as well as seizure-associated differences in hippocampal granule cell activation. Surprisingly, hippocampal granule cell activity as measured by c-*fos* expression after seizure episodes in *CACNA2D2* KO mice differed remarkably after two different seizure-inducing stimuli, gentle handling and subthreshold PTZ injections. Presumably, these differences relate to different mechanisms underlying the behavioral and cellular responses to two different seizure-provoking stimuli and likely involve distinct patterns of neural circuit activation in association with seizure events and altered kinetics of immediate early gene expression after activity.

### Reduced threshold for hippocampal activation in the absence of the α2δ-2 protein

*CACNA2D2* KO mice manifest tonic–clonic seizures in response to doses of PTZ that are subthreshold for WT mice (Ivanov et al., 2004), and we now confirm that a subthreshold dose of PTZ also substantially increases hippocampal granule activity in KO mice when compared with WT mice. The reduced ability to maintain network homeostasis when challenged with proconvulsant stimuli, combined with no change in baseline levels of c-*fos* activation in the dentate, suggests potential dysfunction in inhibitory feedforward networks that maintain network quiescence during periods of enhanced circuit activity in KO mice. Since one of the major interneuron subtypes mediating this form of inhibition, PV+ interneurons, is not diminished in density, a plausible explanation for this phenotype could involve PV+ neuron dysfunction (without a clearly associated gross structural phenotype) or alterations in the excitatory drive of PV+ neurons. Differences in the functional recruitment of PV+ neurons could potentially be investigated by staining tissue from WT and KO mice to assess expression of activity-dependent c-*fos* or Δ*FosB* in PV+ cells after low-dose PTZ. However, even if KO mice appeared to have normal levels of PV+ interneuron activation during seizures, deficiencies in PV+ output connectivity or synaptic transmission could still compromise PV+-mediated inhibition in a manner that would not be detected immunohistochemically and thus would likely require electrophysiological assays.

α2δ-2 was initially identified as a VGCC subunit, suggesting that α2δ-2-expressing cells, such as hippocampal PV+ cells (Cole et al., 2005), might manifest functional changes in both Ca^++^ signaling and Ca^++^-dependent subcellular processes that are not reflected in structural analyses. Indeed, cerebellar Purkinje cells, which highly express α2δ-2 in WT mice, demonstrate profound functional differences in both their afferent synaptic input and endocannabinoid-mediated signaling in the absence of the α2δ-2 protein (Wang et al., 2013; Beeson et al., 2020, 2022). Similarly, neurons lacking the α2δ-1 isoform have altered neurotransmitter release phenotypes (Hoppa et al., 2012), which might be recapitulated in cells that rely on α2δ-2 for similar functions. Either cell intrinsic (action potential waveform) or synaptic (GABA release or excitatory innervation) dysfunction in PV+ cells could cause seizures without grossly apparent structural phenotypes and become behaviorally apparent during conditions of enhanced circuit activation (e.g., PTZ). Additionally, behavioral phenotypes could also become more prominent as circuits mature during mouse adolescence/weaning, when afferent connectivity into the hippocampus has reached adult levels. As genetic epilepsy syndromes can involve functional (electrophysiological) deficits without clear structural correlates (Meisler et al., 2001), hippocampal function in *CACNA2D2* KO mice could be significantly compromised without structural changes in a similar manner.

### Altered patterns of activity-dependent gene expression in the dentate during handling-induced convulsions in *CACNA2D2* KO mice

Surprisingly, *CACNA2D2* KO mice had reduced c-*fos* expression in dentate granule cells in association with handling-induced convulsions, initially suggesting that activity in the hippocampus had decreased below basal levels during these seizure events. In these same mice, however, the expression of the activity-dependent protein *ΔFosB* was markedly increased in KO mice after handling, suggesting that the dentate gyrus actually experienced substantially increased activity during these handling-induced convulsions. As *ΔFosB* expression can actively inhibit c-*fos* expression (Lamothe-Molina et al., 2022), these results suggest that *ΔFosB* might increase quickly during handling-induced convulsions in the KO mice and perhaps have an exaggerated effect on c-*fos* suppression. However, our studies here are unable to determine whether the two observations (*ΔFosB* increase and c-*fos* decrease) are causally related or alternatively whether they represent two different patterns of immediate early gene response to the same stimulus. Additionally, other activity-dependent compensatory mechanisms could produce a form of peri-ictal depression of c-*fos* expression in the hippocampus, similar to prior descriptions of postseizure reductions in cellular activation in the brain (Morgan et al., 1987). As mice continue to have low-grade convulsive activity for the hour after handling, it is also unclear whether handling-associated seizure activity in these mice is being maintained by activity in other regions of the brain or whether dentate granule cell firing persists in the absence of c-*fos* expression, as these data are clearly different between the two different seizure assays examined.

Prior observations provide some potential insights into other brain regions that might be involved in seizures in *CACNA2D2* KO mice. Early histopathological studies of α2δ-2 mutant (*ducky*) mice identified cerebellar, hindbrain, and spinal cord abnormalities, including Purkinje cell atrophy, reduced volumes and cell sizes in brainstem nuclei, and axonal lesions in several spinal cord motor pathways (Meier, 1968). A future brain-wide comprehensive analysis of posthandling c-*fos* expression could elucidate whether any of these regions might have been involved in posthandling seizures. A more recent analysis identified reduced neocortical size and reduced cortical layer V thickness with increased cell density in *ducky* mice (Geisler et al., 2021). As this cortical layer contains pyramidal cells that both receive inputs from and project to the thalamus, it may well be the anatomical substrate for the electroencephalographic SWDs characteristic of homozygous mutant mice (Barclay et al., 2001). However, as these SWDs are characteristic of “absence”-type seizures typically associated with behavioral arrest, rather than motor convulsions (Barclay et al., 2001), it remains unclear whether the observed motor convulsions in *CACNA2D2* KO mice are the result of aberrant thalamocortical activity or due to circuit dysfunction elsewhere in the central nervous system. Alternatively, as other mutant mouse strains, such as the SWD-prone *stargazer* mutant mouse, can also have motor seizure manifestations and dentate gyrus histopathology (Noebels et al., 1990; Qiao and Noebels, 1993), *CACNA2D2* KO mice might represent another strain with SWDs that intermittently manifest motor convulsions and neural seizure activity outside of the thalamocortical circuit.

One unexpected difference uncovered in our study was a small but statistically significant increase in the density of calretinin-positive hilar mossy cells. Mossy cells preferentially drive inhibitory circuit function and can control seizure activity (Bui et al., 2018). Thus, an increased density of functional mossy cell outputs could potentially reduce granule cell activity levels in KO mice, if convulsive seizures originate outside of the hippocampus and increase inhibitory circuit activity through an enhanced mossy cell network. Thus, increased mossy cell density might provide a small amount of functional compensation to the hyperexcitable dentate. However, given the small absolute magnitude of the increased hilar mossy cell density, it is unclear whether this small increase would substantially alter functional output of this pathway, as mossy cells project widely to bilateral hippocampus and might homeostatically adjust their output connectivity accordingly (Butler et al., 2022).

### Future directions

We performed our analyses at 1 month of age, which correlates with the onset of behavioral seizures noted in mice lacking the α2δ-2 protein (Meier, 1968; Ivanov et al., 2004). The lack of several histopathological markers of TLE at this time indicates that the circuit rearrangements that they represent (recurrent mossy fiber sprouting, loss of hippocampal neuron populations, glial activation) are not required for mice to express seizures or increased excitability at this age.

Although behavioral seizures do not require global dentate gyrus activation in the hippocampus at this early timepoint, seizures can lead to secondary changes in circuit structure and progressively worsening disease (Cavazos et al., 1991). Thus, our analysis does not preclude potential hippocampal involvement in the progression and worsening of epilepsy as mice continue to age, which might include changes in hippocampal structure in adult mice. Improvements in animal husbandry that allow for enhanced survival of KO mice into adulthood (Geisler et al., 2021) could facilitate analysis at these later stages.

An inherent limitation to our study is the restriction of our analysis to a limited number of histologic assays. For example, while our focus on PV+ interneurons was derived in part from the expression of the α2δ-2 protein in this cell type, other interneuron types, such as somatostatin-expressing interneurons, also decrease in density in certain forms of epilepsy (Santhakumar et al., 2000; Hofmann et al., 2016) and were not examined here. Finally, electrophysiological studies could elucidate any changes to intrinsic hippocampal circuit function, as well as any potential alterations in afferent connectivity into the hippocampus, that are not apparent on immunohistological analysis or might not have been evident given our explicit focus on the dentate gyrus.

Future work will hopefully determine whether restoration of the α2δ-2 function later in life, perhaps through viral vector-mediated rescue approaches, would be sufficient to diminish behavioral seizures in the face of seizure-related circuit remodeling. This could be performed experimentally in a region-specific manner, which would both help elucidate mechanisms of seizure initiation and also potentially be relevant to families with α2δ-2 mutations or other epilepsy-associated channelopathies.

## Synthesis

Reviewing Editor: Viji Santhakumar, University of California Riverside Decisions are customarily a result of the Reviewing Editor and the peer reviewers coming together and discussing their recommendations until a consensus is reached. When revisions are invited, a fact-based synthesis statement explaining their decision and outlining what is needed to prepare a revision will be listed below. The following reviewer(s) agreed to reveal their identity: NONE.

The study examined mice that lack α2δ2 voltage-gated calcium channel (KO) for neuronal activity using labeling for the activity-dependent immediate early gene, c-fos and various histopathological changes associate with temporal lobe epilepsy. The underlying rationale is that α2δ2 loss of function is associated with epilepsy in animal models and humans. Mice were examined at the age of seizure onset in this model. Although the mice exhibit spike wave discharges suggesting thalamocortical seizure, the study focused on the dentate gyrus. They find that no difference in basal c-fos expression between KO and wild type mice. However, although subthreshold PTZ increased c-fos labeling in KO mice, handling, which is known to induce seizures in these KO mice, paradoxically decreased c-fos expression in KO mice. Notably, glial activation (GFAP, Iba1), neurogenesis (DCX) and mossy fiber sprouting (ZnT3), CA3 and PV neuron density were not different between genotypes. Unexpectedly, mossy cell density in KO mice appeared greater than controls. While the study appears to be carefully implemented, the focus on the dorsal dentate gyrus is a concern.

1. Expression of c-fos was examined in dorsal dentate gyrus and differences may or may not occur in ventral hippocampus which often shows more extensive engagement and cell loss in epilepsy models. Thus, examination of more ventral sections is warranted. This is especially relevant to baseline measurements (homecage) that lacked significant differences between KO and control mice. This could also clarify and validate the differential effects seen with handling and PTZ.

2. Regarding the PTZ data, they appear to cluster in 2 groups. It appears that the PTZ the severity (max racine) did not correlate with a weak vs strong increase in GC cFos. Some clarification on the methods is needed. During the hour that the mice were monitored, did the frequency of seizures or duration of seizing correlate with cFos labeling? Could the number of prior seizures explain the bimodal cFos labeling? I.e. in the pilo model of TLE Peng and Houser (2005) found little cFos activation in mice that experienced multiple behavioral seizures in the previous 24h vs robust activation in mice with a longer seizure interval, making frequency quite relevant (are behavioral seizures rare/frequent/highly variable?).

3. Related to the above, it should be noted that cFos labels recently active cells while DeltaFosB is expressed in chronically activated cells and cFos expression can be decreased in chronically active cells (doi: 10.1038/s41467-022-33947-w). This issue needs to be discussed. Additionally, the statement in the results (page 11) "To assess whether this phenotype was associated with chronically elevated neuronal activity..." needs to be revised accordingly.

4. While authors suggest future brain-wide comprehensive analysis of cFos expression, the available coronal sections should be examined for baseline and handling-induced cFos expression in the nRT. It would also be very helpful to show lower magnification images of cFos labeling that include the whole hippocampus, in Figure 1.

5. The reasons for potential increase in mossy cell density in KO mice need to be discussed. Shrinkage evidenced by cell loss or gliosis, reduced granule cell neurogenesis, were ruled out. Would normalizing MC counts to GC counts (as for cFos and PV), or increasing the sampling, resolve the discrepancy? Perhaps it is an artifact of the samples.

6. Seizure severity between PTZ and handling seem different. Comparison of Fig 1E and 2B indicates that the handling-induced seizures were less severe vs PTZ ones. Is that the case? Also, was the latency to first seizure different between the 2 approaches? Whether this could explain opposing effects on c-fos expression should be discussed.

7. Several methodological aspects need clarification.

a. it is unclear which septotemporal level was used for the analysis and if this was consistent between groups. Please clarify.

b. Exclusion criteria by which ".. small CR+ somata in the subgranular zone that were most likely immature granule cell neurons," were excluded need to be specified in the methods.

c. To quantify immunostaining, it is indicated that only a subset of the tissue was sampled. Was this because some primaries did not sufficiently penetrate the 100 um sections during overnight incubations at 4 degrees Celsius that single images (Calretinin, Dcx, Iba1) or partial stacks (GFAP) rather than full stacks (PV, Ki67, Mac-2) were analyzed for some markers? The number of (dorsal) images or stack per mouse used to quantify markers (apart from cFos) should be clear in the methods.

d. Please indicate how CA3 pyramidal cell density was quantified using DAPI in light of many overlapping somata in the CA3 pyramidal layer?

8. It is mentioned that there were no sex differences but data is not shown. Please include which comparisons included sex as a variable and outcomes of each test.

9. Given the focus on the dentate, most references to the 'hippocampus/hippocampal' should be restricted to the (dorsal) 'dentate gyrus.'

10. The text on p. 15 and Figure 2 legend reports 9 mice (WT & KO, respectively) for the PTZ experiment, but only 8 dots are apparent in the graph for each genotype. Additionally, the text reports 0 WT scored Racine {greater than or equal to} 3, but Figure 2B shows one scoring 4.

11. Page 20: it would be better to phrase as "The PV+ cell density in both regions was not different between WT and KO mice (Figures 6A-C)."

## Author Response

Synthesis Statement for Author (Required): The reviewers concur that the additional staining and analysis undertaken by the authors as well as the revisions have strengthened the manuscript. A few additional clarification were recommended. Thank you for your additional comments; please see our responses below. Changes to the manuscript are referenced in bold text. 1. The new data showing potential differences (p = 0.055) in ΔFosB labeling between PTZ in WT vs α2δ-2 KOs. It would be interesting to evaluate if ΔFosB expression correlates with the bimodal cFos labeling following PTZ in KOs? Could the authors comment on whether ΔFosB following PTZ showed bimodal distribution? Is it possible that KOs with low cFos have high ΔFosB? This is an excellent question, and a solid hypothesis for why some mice had low c-fos expression after clear PTZ-induced behavioral seizures. Specifically, given our finding that handing-induced convulsions in KO mice had below-baseline c-fos expression associated with high ΔFosB expression, the reviewers wondered whether some of the PTZ-treated KO mice may have had similarly increased ΔFosB expression after PTZ-induced seizures with an associated decrease in c-fos expression. To address this question, we compared relative c-fos and ΔFosB expression for each mouse in the cohort of KO mice that received PTZ. Interestingly, although ΔFosB staining intensity did have a bimodal distribution in KO mice, there was no obvious relationship between ΔFosB and c-fos staining. There were 4 mice in the PTZ group that had a substantial increase in ΔFosB staining intensity (~4 fold above WT) and 4 (of the 8) mice that were fairly similar to WT controls, but these only partially overlapped with the "high c-fos" mice. To assess this statistically, we performed a simple linear regression. For the KO mice, there was no significant correlation between c-fos+ cell density and ΔFosB (r2 = 0.11, slope 0.002, F= 0.73, p = 0.42) indicating that these values were neither correlated or anti correlated. In contrast, the four mice with high dorsal c-fos+ cell density also had high ventral c fos+ cell density, and the correlation between dorsal and ventral c-fos expression was highly significant (r2 = 0.75, slope 0.101, F= 18.1, p = 0.005), suggesting that c-fos expression increase somewhat uniformly across the hippocampus in a subset of mice after PTZ. These additional analyses were added to the manuscript. Although this is a bit unsatisfying, it harkens back to the observation that the bimodal increase in c-fos expression after PTZ was not statistically related to seizure intensity either. This suggests that there may either be several different hippocampal activation patterns after low-dose PTZ, or that the timepoint of our analysis (1 hour after PTZ) represents a stage at which gene expression is dynamic, such that small variations in timing between mice could alter the findings. It also suggests that patterns of IEG expression might be pan-hippocampal, since c-fos increased globally throughout the hippocampus to a similar degree in each mouse. 2. Could the authors comment on whether Mac-2 representative image was not shown because of sparse labeling? Very sparse labeling is precisely the reason these images were not shown. The entire tissue section was imaged using a lower power objective to obtain confocal Z-stacks, in an attempt to maximize the tissue volume sampled for Mac2+ quantification. Even with this strategy, each stack contained (on average) fewer than 3 Mac2+ cells. When displayed in two dimensions as a low-power projected image stack, it would difficult to see the small cells without very large format figures, so images were not shown. We added some text to the results noting the small number of cells typically seen, in addition to the comment already in the Methods section about the strategy used to quantify the low density of Mac2+ cells in each group. Importantly, there was no difference in Mac2 staining between groups, suggesting no dramatic differences in microglial activation between genotypes. 3. The term "up to 4" sections were acquired for each experiment is confusing. Was the maximum 4 and were fewer used? What was the lower limit -please clarify. If most studies included 4 sections and if less than 4 were used in a few experiments, it would help to state that 'generally' 4 sections were analyzed for each experiment and indicate exceptions to this.. Apologies for the confusion. Four dentate images were taken from each mouse, but a small number.
